# Supplementary figures and images for: Coffee consumption and risk of breast cancer: A Mendelian randomization study
Source: PLoS One. 2021 Jan 19;16(1):e0236904. doi: 10.1371/journal.pone.0236904 (PMC7815134; doi:10.1371/journal.pone.0236904)

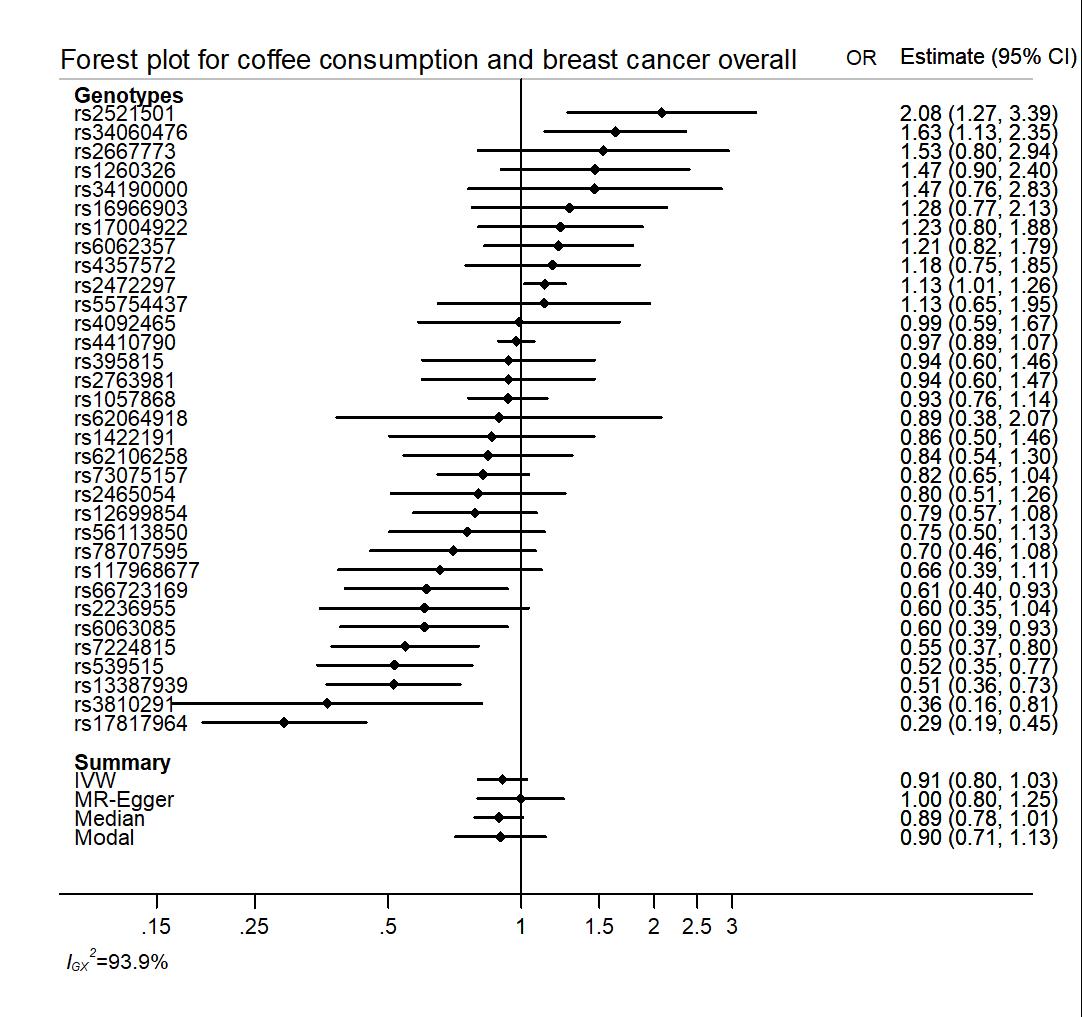

Supplement: S1 Fig — (JPG) [file pone.0236904.s001.jpg]

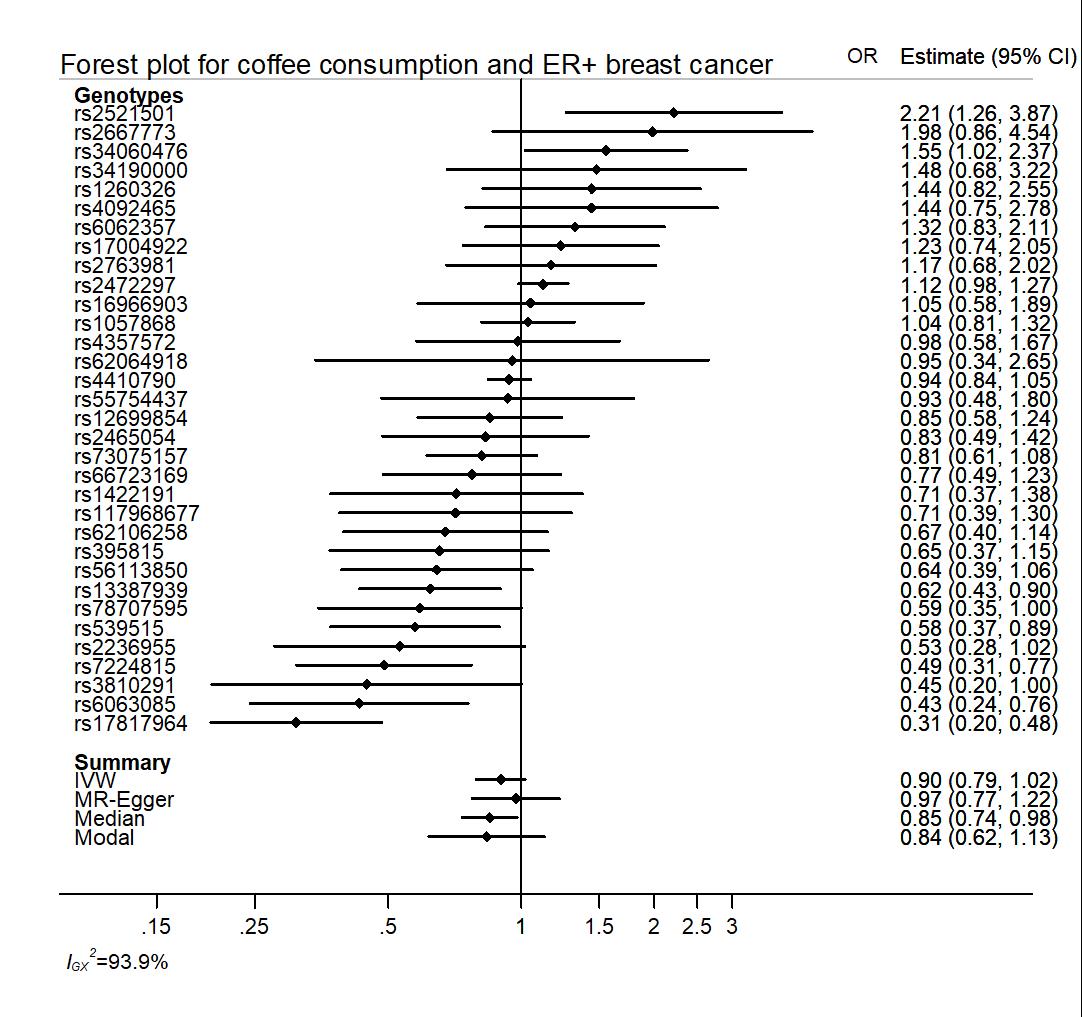

Supplement: S2 Fig — (JPG) [file pone.0236904.s002.jpg]

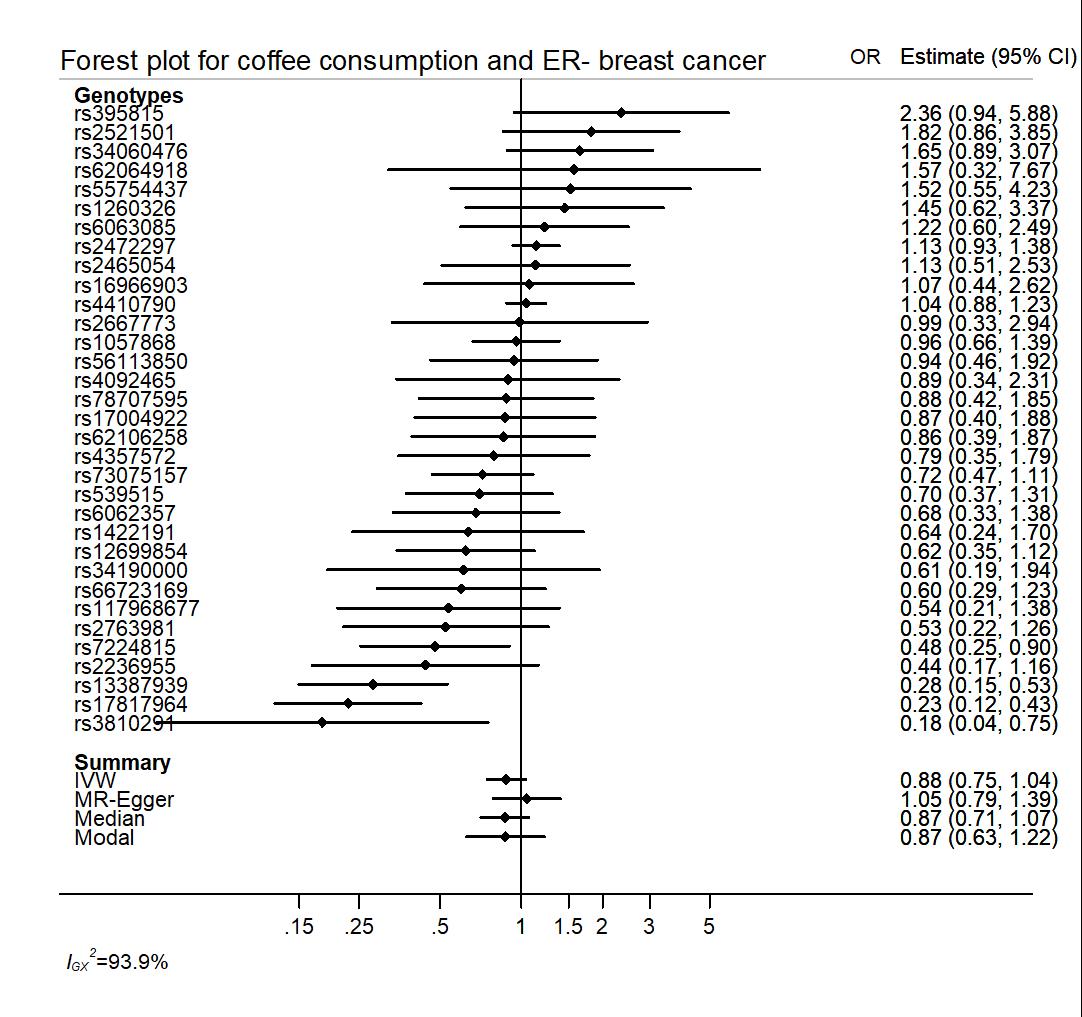

Supplement: S3 Fig — (JPG) [file pone.0236904.s003.jpg]

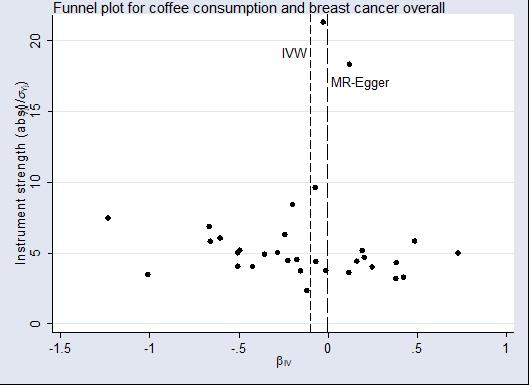

Supplement: S4 Fig — (JPG) [file pone.0236904.s004.jpg]

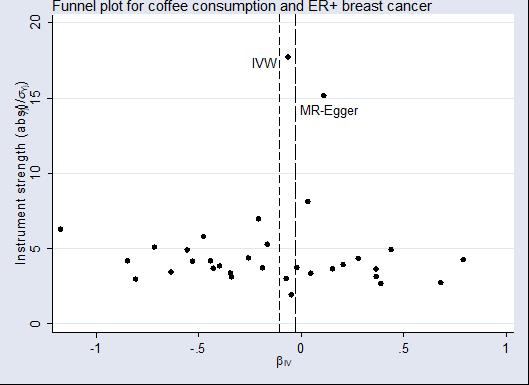

Supplement: S5 Fig — (JPG) [file pone.0236904.s005.jpg]

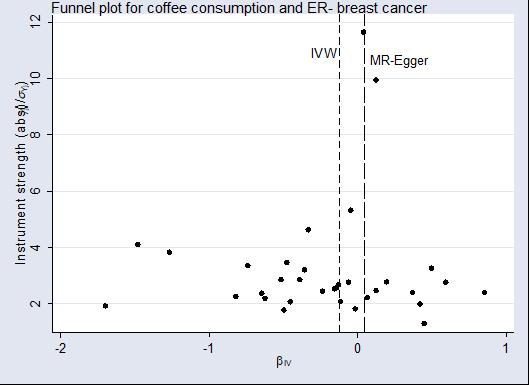

Supplement: S6 Fig — (JPG) [file pone.0236904.s006.jpg]
