# Supplementary material for: Coffee consumption and risk of breast cancer: A Mendelian randomization study
Source: PLoS One. 2021 Jan 19;16(1):e0236904. doi: 10.1371/journal.pone.0236904 (PMC7815134; doi:10.1371/journal.pone.0236904)
Supplement: Attachment — Submitted filename: Response to Reviewers.docx [file pone.0236904.s010.docx]

**RESPONSES TO REVIEWERS -PONE-D-20-21709**
*Coffee consumption and risk of breast cancer: a Mendelian Randomization study*

**Editor:**

#1 I would ask to the authors to specify all the parameters involved in the computation of the study power (page 7), including the type I error. It’s also not clear to the reader what parameters 0.89, 0.87 and 0.80 represent, i.e. regression coefficients? Or what else?

*Response: We have specified the parameters in the computation of the study power under the heading Statistical power page 7, including the type I error rate.*

#2 Revised financial disclosure

*Response:* *This work was supported by the World Cancer Research Fund International Regular Grant Programme (WCRF 2014/1180 to Konstantinos K. Tsilidis). RMM was supported by a Cancer Research UK (C18281/A19169) programme grant (the Integrative Cancer Epidemiology Programme) and is part of the Medical Research Council Integrative Epidemiology Unit at the University of Bristol supported by the Medical Research Council (MC_UU_12013/1, MC_UU_12013/2, and MC_UU_12013/3) and the University of Bristol. RMM is also supported by the National Institute for Health Research (NIHR) Bristol Biomedical Research Centre which is funded by the National Institute for Health Research (NIHR) and is a partnership between University Hospitals Bristol NHS Foundation Trust and the University of Bristol.*

We would be grateful if you could change the relevant online submission form on our behalf.

#3 Revised ethics statement

*Response:* *Not applicable, as only publicly available summary association data were used.*

**Reviewer #1**: 

#1 What kinds of subtypes did you assess? I understand that you investigated the relationship between genetically predicted coffee consumption and risk of breast cancer overall as well as breast cancer subtypes incorporating several MR methods to assess the impact of potential MR assumption violations. However, there were no information about subtypes you assessed in method. There are several subtypes in breast cancer, so please state which subtypes you assessed in method section.

*Response*: *Thank you. We have added more information about the estrogen receptor positive and negative breast cancer in the Methods section (Genetic data on breast cancer).*

#2 Have you considered the dose-response analysis, especially for the postmenopausal women? There is a dose-response meta-analysis regarding the association between coffee intake and breast cancer risk. (Nutrients. 2018 Jan 23;10(2):112) The result showed that consumption of four cups of coffee per day was associated with a 10% reduction in postmenopausal cancer risk. If you have done, please include it in the results.

*Response:* *We have already performed analyses* *per 1 cup per day of genetically predicted coffee consumption. We are not able to run analyses only on postmenopausal women since the available GWA studies on breast cancer presented information on overall breast cancer, but the majority (85%) of the included women were postmenopausal. We have acknowledged this issue in the limitation section of the Discussion.*

#3 You mentioned that you have considered some potentially important confounders of coffee and breast cancer association (BMI, age at menarche, alcohol, smoking, and age at menopause). How did you choose these factors? There are more factors potentially associated with breast cancer, as parity, age at first birth, family history of breast cancer, and use of menopausal hormone therapy. (Biochim Biophys Acta. 2015 Aug;1856(1):73-85) Is it possible to consider these factors in the analysis?

*Response: We ran sensitivity analyses excluding SNPs in our genetic instrument associated with risk factors for breast cancer to probe into potential horizontal pleiotropy. The reason for not excluding SNPs associated with parity, age at first birth or HRT use was that relevant GWA studies have not been performed or the SNPs in our instrument were not associated with these risk factors.*

**Reviewer #2**: 
#1 Given substantial heterogeneity in ratio estimates between variants, it is misleading to present results from the fixed IVW analyses. I suggest to remove “IVW fixed” results from figure 1, 2 and 3.

*Response:* *We have removed the fixed IVW results from figures 1, 2 and 3.*

#2 In the conclusion, authors state “…, but we cannot rule out existence of weak inverse association”. I agree with authors that there could be a weak association which was not picked up by the study, but I do not think it is appropriate to infer directionality of such association.

*Response: We have removed the direction of the weak association both in the conclusion in the Abstract (page 3) and in the Conclusion of the manuscript (page 16). Thank you.*

#3 In the discussion, authors should also acknowledge linearity assumption with their MR analyses. If there is a threshold adverse effect, this could have been masked by fitting a linear model.

*Response: We have acknowledged the linearity assumption issue in the limitation section of the Discussion.*
